# Prenatal Bisphenol B Exposure Induces Adult Male Offspring Reproductive Dysfunction via ERα Inhibition-Triggered MHC I-Mediated Testicular Immunological Responses

**DOI:** 10.3390/toxics13060423

**Published:** 2025-05-22

**Authors:** Nannan Chen, Xiaotian Li, Shenrui Zhou, Xin Peng, Senlin Xue, Yuetong Liu, Tingwang Jiang, Wei Yan

**Affiliations:** 1 School of Life Science, Xuzhou Medical University, Xuzhou 221004, China; 303112111087@stu.xzhmu.edu.cn (N.C.); 202123010127@stu.xzhmu.edu.cn (X.L.); 202123010121@stu.xzhmu.edu.cn (S.Z.); 202123010105@stu.xzhmu.edu.cn (X.P.); xuesenlin2020@163.com (S.X.); 2 Department of Key Laboratory, Affiliated Changshu Hospital of Nantong University, Changshu 215500, China; 5301002@ntu.edu.cn; 3 School of Nursing, Xuzhou Medical University, Xuzhou 221004, China; 202304011321@stu.xzhmu.edu.cn

**Keywords:** maternal BPB exposure, male reproductive dysfunction, testicular immunity, MHC I, ERα

## Abstract

As an emerging endocrine-disrupting agent and structural analog of bisphenol A (BPA), bisphenol B (BPB) raises significant concerns due to its potential to induce male reproductive toxicity. Despite its presence in maternal bodily fluids, the effects of BPB exposure on the reproductive system and its mechanisms in adult male offspring are poorly understood. By establishing a maternal BPB exposure model in mice, we found that the exposure reduced the relative weights of seminal vesicles and preputial glands, decreased the thickness of the seminiferous epithelium, enlarged the lumen area of seminiferous tubules, and lowered testosterone concentration and synthesis, as well as sperm count in 10-week-old male offspring. Bioinformatic analyses revealed that the differentially expressed genes were significantly associated with major histocompatibility complex I (MHC I)-mediated immunological processes, including immune system processes, antigen processing and presentation of exogenous peptide antigens via MHC class I, and interleukin-2 production. Importantly, molecular docking proposed a potential mechanistic model wherein BPB bound to estrogen receptor α (ERα) suppressed its testicular expression and triggered MHC class I gene overexpression, potentially promoting macrophage infiltration, CD4+/CD8+ T cell activation, and pro-inflammatory cytokine production. Our findings provide critical insights into the adverse effects of maternal BPB exposure on male reproductive development, suggesting that impairments in testicular morphology and spermatogenesis may be attributed to MHC I-mediated immunological responses and hormonal imbalances resulting from inhibited ERα signaling. These results underscore not only the toxicological risks associated with BPB but also potential therapeutic targets for mitigating male reproductive dysfunction.

## 1. Introduction

Bisphenol A (BPA) has been widely used to make polymers like polycarbonate plastics, epoxy resins, and thermal paper, and it is commonly found in everyday consumer products, including plastic items, receipts, and food packaging [1]. Recognized as an endocrine disruptor, BPA has led numerous countries and regions to implement regulations restricting its use [2]. Consequently, several structural analogs have gradually supplanted BPA, introducing various substitutes into the environment, with BPB being a notable example [3] which shares a strong structural similarity with BPA but differs only by an additional methyl group on the central carbon (Figure S1) [4].

BPB, which has been approved by the U.S. Food and Drug Administration (FDA) for use as an indirect food additive in resins and polymer coatings, has been detected in environmental media, food products, consumer goods, and human urine and blood, highlighting its multiple pathways of exposure [5]. Comparative serum analyses revealed that while all 11 healthy control subjects were free from bisphenols, 30 out of 51 patients with endometriosis exhibited detectable levels of BPA, and 27 patients (6.58%) had detectable BPB, with an average concentration of 0.20 ng/mL [6]. Furthermore, biological monitoring indicated analogous concentrations of BPB and BPA in urine samples obtained from Portuguese volunteers [7]. Serum analyses conducted across 10 provinces in China indicated the highest concentrations of BPB in Shandong (median 0.906 ng/mL), followed by Shanxi (0.776 ng/mL) and Hebei (0.763 ng/mL) [8]. Additionally, a review on bisphenol exposure in pregnant or nursing women revealed BPB levels surpassing BPA, with concentrations as high as 5.15 ng/mL [9]. Despite being less analyzed and reported than other BPA alternatives, such as BPF and BPS, BPB’s higher endocrine activity [10], stronger estrogenic effects [11], greater resistance to biodegradation, and higher propensity for accumulation in organisms raise substantial concerns regarding its environmental and health risks [12].

Due to its strong estrogenic, androgenic, and anti-androgenic activities comparable to those of BPA [5], BPB has been extensively studied for its toxic effects, particularly its reproductive toxicity. In experimental rat models, both 28-day and 48-week BPB exposure revealed notable perturbations in spermatogenesis, a diminished height of the seminiferous tubules, alterations in various sperm parameters, and a reduction in the weight of the testes, epididymis, and seminal vesicles. Correspondingly, research conducted on zebrafish over a 21-day exposure period highlighted a decrease in egg production, lower hatching success rates, and a decline in embryo viability [4]. Such findings underscore a significant disruption of male reproductive functions attributable to BPB exposure. Furthermore, BPB has been identified in maternal serum and placental tissues [13], which raises substantial concerns given the critical nature of pregnancy and lactation for the reproductive development of offspring. Recent investigation has indicated that prenatal exposure to BPB substantially reduces sperm production, impairs histological structures within the testis and epididymis, and alters antioxidant enzyme levels, plasma testosterone, and estrogen concentrations in male offspring [14]. Nevertheless, the precise pathological mechanisms underlying these adverse effects remain inadequately elucidated.

To address this gap, we established an animal model to investigate the effects of maternal BPB exposure on the reproductive system development in male mouse offspring. By integrating phenotypic detections, histological observations, and mechanistic analyses, we aimed to uncover how maternal BPB exposure impacts reproductive organ development, testicular morphology, sperm production, and hormone levels in adult male offspring and elucidate the pathological mechanisms related to testicular immune abnormalities mediated by estrogen receptor signaling. This research offers vital toxicological insights into the reproductive developmental consequences of BPB exposure and contributes significantly to the assessment of BPB’s safety while also identifying potential therapeutic targets for male reproductive dysfunction.

## 2. Materials and Methods

### 2.1. Animals and Maternal BPB Exposure

Eight-week-old ICR mice were purchased from Vitality River Laboratory Animal Technology Co., Ltd. (Beijing, China) and maintained in an SPF animal facility. Male and female mice were housed together in a cage at a 1:2 ratio following one week of acclimatization. Pregnancy was confirmed by vaginal plug detection the following morning. The study was authorized by the Research Ethics Committee of Xuzhou Medical University (approval number: 202404T025). BPB, with purity higher than 98.0%, was purchased from Tokyo Chemical Industry Co. Ltd. (Tokyo, Japan) and dissolved in corn oil. In this study, pregnant mice received oral administration of BPB (300 µg/kg body weight, N = 7) or corn oil (vehicle group, N = 6) every other day from gestational day 0.5 until delivery. Testicular tissues were collected on postnatal day (PND) 70 for further analysis.

The rationale for selecting the exposure dose in this study is based on the following considerations: (1) Existing studies have documented serum BPB concentrations of 0.20–0.906 ng/mL in human populations [7,8]. However, the pharmacokinetic data for BPB were lacking. Based on the comparable uptake and depuration kinetics between BPB and BPA [15], we referenced BPA exposure data to estimate potential human exposure levels of BPB. Vandenberg et al. systematically reviewed BPA concentrations in human tissues and demonstrated that daily oral doses of ≥500 μg/kg BPA are required to achieve serum concentrations (0.3–4 ng/mL) observed in adults [16,17]. Supporting this, Taylor et al. reported that a 400 µg/kg oral dose produced 24 h serum concentrations of 0.5 ng/mL in both primates and rodents [18]. Through pharmacokinetic modeling accounting for species-specific metabolic rates and body surface area differences, they estimated human equivalent doses (HEDs) of 0.1–86 mg/kg/day (mean: 2.4–9 mg/kg/day). Importantly, multiple biomonitoring studies indicate that achieving blood concentrations of 1 ng/mL (within reported human detection ranges) requires oral doses of 10–400 μg/kg in rodents and non-human primate models [19]. Given that detected BPB levels in human samples are slightly below 1 ng/mL, the 300 µg/kg dose was selected to maintain biological relevance. (2) This dosing regimen aligns with recent investigations demonstrating that maternal BPB exposure at 300 µg/kg every other day induces measurable developmental toxicity, including impaired placental vascularization and glucose dysregulation in offspring [20]. The established protocol ensures comparability with emerging toxicological data on bisphenol analogs. Based on the aforementioned rationale, the exposure concentration utilized in this study was scientifically justified and could mimic real-world exposure scenarios.

### 2.2. Body Weight and Reproductive Organ Indices Test

No mortality was observed among the offspring. On PND 70, the male offspring were weighed, and their reproductive organs—such as the testis, seminal vesicle, preputial gland, and epididymis—were separated and analyzed. Additionally, the anogenital distance (AGD) and the AGD index (AGI), which is calculated as AGD divided by the body weight, were assessed concurrently.

### 2.3. Histological Analyses

Testicular tissues were harvested from male offspring mice at the age of 70 days and then fixed in 4% paraformaldehyde solution at 4 °C overnight. The samples were dehydrated using a graded series of alcohol, cleared with xylene, and then embedded in paraffin wax. Next, 5 μm thick sections were cut from the embedded tissue and stained with hematoxylin-eosin (H&E) for histological examination. After sealing with neutral balsam, morphological changes were observed using an Olympus BX53 microscope (Tokyo, Japan). At least three mice from both the BPB and control groups were selected for statistical analysis. Each mouse was examined with 5 to 7 tissue slices. Parameters measured included testicular tubule diameter, lumen area, epithelial thickness, and the number of germ cells. Measurements were performed on over 30 round or nearly round seminiferous tubules using ImageJ software (ImageJ 1.54i). For seminiferous tubule diameter measurement, two perpendicular lines were drawn across the tubule center, and the average length of these lines was calculated as the diameter. For seminiferous epithelium thickness, measurements were taken from the basal layer to the inner membrane where germ cells appear, and each tubule was measured twice to obtain an average value.

### 2.4. Sperm Count Test

The right epididymis from 70-day-old male offspring mice was dissected into small fragments and immersed in 0.5 mL of PBS preheated to 37 °C (pH 7.4) to facilitate sperm release. Following a 30 min incubation, 10 µL of the mixture was aliquoted from each sample to determine sperm count, and results were reported as 10^6^/mouse.

### 2.5. Hormone Measure

Blood samples collected from 70-day-old male offspring mice were stored at 4 °C before centrifugation to isolate serum for hormone analysis. Serum testosterone levels were measured using the UniCel DxI 800 Access Immunoassay System (Beckman Coulter, Danvers, MA, USA), which performs chemiluminescent immunoassays.

### 2.6. Total Testicular RNA-seq and Data Analysis

RNA-seq analysis was performed by Shanghai Biotechnology Corporation (Shanghai, China), which provided gene expression profiles of testicular tissues from mice exposed to BPB and Vehicle on PND 70 and identified differentially expressed genes (DEGs). Sequencing was conducted on the Illumina HiSeq 2500 platform, and gene expression levels were quantified using the FPKM method, as detailed in our previous study [21]. Genes with a |fold change| ≥ 1.5 and a *p*-value < 0.05 were considered statistically significant. The biological functions of the DEGs were assessed using Gene Ontology (GO) classification, as well as KEGG pathway enrichment analysis, both performed via the DAVID online tool (https://davidbioinformatics.nih.gov/).

### 2.7. RNA Extraction and Reverse Transcription PCR

Testes from 70-day-old male offspring were collected, flash-frozen in liquid nitrogen, treated with Trizol reagent, and homogenized to extract total RNA. cDNA was synthesized following the manufacturer’s instructions for the reverse transcription kit (Bio-Rad, Hercules, CA, USA) using 1 µg of total RNA as the template for qRT-PCR. Primers listed in Table 1 were procured from Sangon Biotech Co., Ltd. (Shanghai, China). Relative expression of the target gene, based on SYBR Green detection, was calculated as the ratio of the sample to the control, normalized to the reference gene *Gapdh*, and analyzed using the 2^−ΔΔCt^ method.

### 2.8. Immunostaining and Analyses

Testicular tissues were fixed in 4% paraformaldehyde for 12 h, followed by dehydration in 15% and 30% sucrose until the tissue sank, and then embedded. Frozen sections with 5 μm thickness were treated with 3% bovine serum albumin for 1 h to block non-specific binding, followed by a 24 h incubation with the primary antibodies, including anti-TRA98 (#MAB7953, Abnova, Taiwan, China), anti-3β-HSD (#sc-100466, SANTA, Dallas, TX, USA), anti-F4/80 (#DF2789, Affinit, Changzhou, China), anti-CD4 (#158692, Bio-Rad, Hercules, CA, USA), and anti-CD8 (#154984, Bio-Rad, Hercules, CA, USA) diluted at 1:200. Following incubation with secondary antibodies, including CoraLite488-conjugated Goat Anti-Mouse IgG (H+L) (1:500; #SA00013-1, Proteintech, Wuhan, China) and CoraLite594-conjugated Goat Anti-Rabbit IgG (H+L) (1:500; #SA00013-3, Proteintech, Wuhan, China) for 1 h, the sections were thoroughly washed with phosphate-buffered saline (pH = 7.4), mounted with anti-fade reagent, and then examined under a fluorescence microscope (Olympus IX71, Tokyo, Japan). Data analyses were performed using ImageJ software (ImageJ 1.54i).

### 2.9. Molecular Docking

Direct interactions between BPB and estrogen receptor alpha (ERα) were analyzed using Discovery Studio 2020 and AutoDock 4.2 software. The three-dimensional structures of BPB were obtained from the PubChem database (https://pubchem.ncbi.nlm.nih.gov/). The X-ray crystal structures of the ERα (ID: 2QZO) were from the Protein Data Bank (PDB, https://www.rcsb.org/) [22]. The hydrogen atoms and charges were added to the proteins and ligands using AutoDock Tools 1.5.7. Docking was performed using PyRx integrated Autodock and AutoDock 4.2 programs. The intermolecular interactions (i.e., hydrogen bonds and others) between BPB and ligands were visualized with PyMOL 3.1.

### 2.10. Western Blot

The testicular tissues were dissected from the mouse pups (PND 70), and protein was isolated RIPA lysis buffer (P0013B, Beyotime, Shanghai, China) with a protease inhibitor. The concentrations of the protein samples were measured using the BCA protein assay kit (P0012S, Beyotime, Shanghai, China). A total of 30 µg of each protein sample was separated via 10% SDS-PAGE and then transferred to nitrocellulose (NC) membranes (Cat#P/N 66485, BioTrace, New York, NY, USA). The membrane was blocked with 5% skim milk in TBST for 1 h at room temperature. After that, the membrane was incubated overnight at 4 °C with Mouse anti-3β-HSD (1:5000, Cat#67572-1-lg, Proteintech, Wuhan, China) and Mouse anti-GAPDH (1:30,000, Cat# 60004-1-Ig, Proteintech, Wuhan, China). Subsequently, the secondary antibody Goat anti-mouse IgG (Cat#A23910, Abbkine, Wuhan, China) diluted at 1:10,000 in washing buffer was applied and incubated for 2 h at room temperature, and then protein expression was visualized utilizing a dual infrared laser imaging system (Odyssey LI-COR, Lincoln, NE, USA). Band intensity was analyzed using ImageJ software.

### 2.11. Statistical Analysis

Data were expressed as mean ± SEM. Statistical analysis was conducted using a two-tailed *t*-test, with a significance threshold of *p* < 0.05 to evaluate differences between the BPB and vehicle groups.

## 3. Results

### 3.1. Maternal BPB Exposure Disrupts Reproductive Organ Indices in Adult Male Offspring

**Figure 1:**
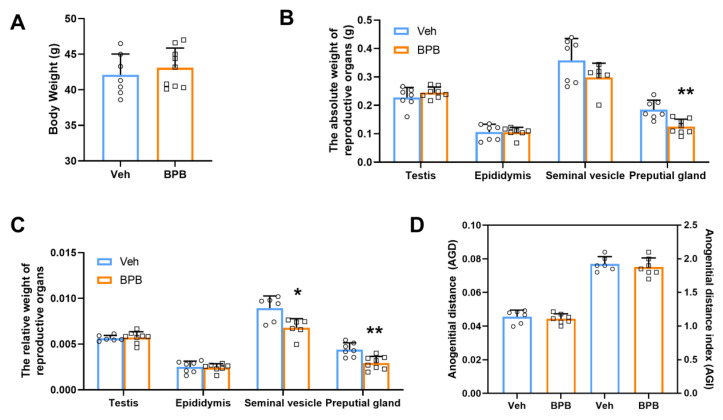
Effects of maternal BPB exposure on the reproductive organs in adult male offspring. (**A**) Body weight; (**B**) absolute and relative (**C**) weights of reproductive organs; (**D**) AGD and AGI. Data are shown as mean ± SEM (N = 6–9). The circles represent individual data points from Veh-exposed mice and squares represent individual data points from BPB-exposed mice. * *p* < 0.05 and ** *p* < 0.01 indicate significant differences between two groups.

To assess the effects of maternal BPB exposure on the development of the male reproductive system in 70-day-old offspring, we evaluated body weight and the weights of reproductive organs (including the testis, epididymis, seminal vesicle, and preputial gland) in prenatal BPB-treated offspring. As illustrated in Figure 1, no obvious changes were observed between the two groups in terms of male body weight (Figure 1A,B) or the absolute weights of the testis, epididymis, and seminal vesicle following prenatal BPB exposure. However, a significant decrease of approximately 33.4% was noted in the weight of the preputial gland (Figure 1B). Furthermore, the relative weights of the seminal vesicles and preputial glands were significantly reduced by approximately 24.1% and 33.2%, respectively (Figure 1C). Additionally, analysis of the AGD—a validated endpoint marker for male reproductive health—revealed that although the differences were not statistically significant, AGD and the AGI decreased to varying degrees after exposure (Figure 1D). These findings indicate that maternal exposure to BPB disrupts sexual organ development, potentially contributing to reproductive impairment in adult male offspring.

### 3.2. Maternal BPB Exposure Impairs Testicular Morphology in Adult Male Offspring

**Figure 2:**
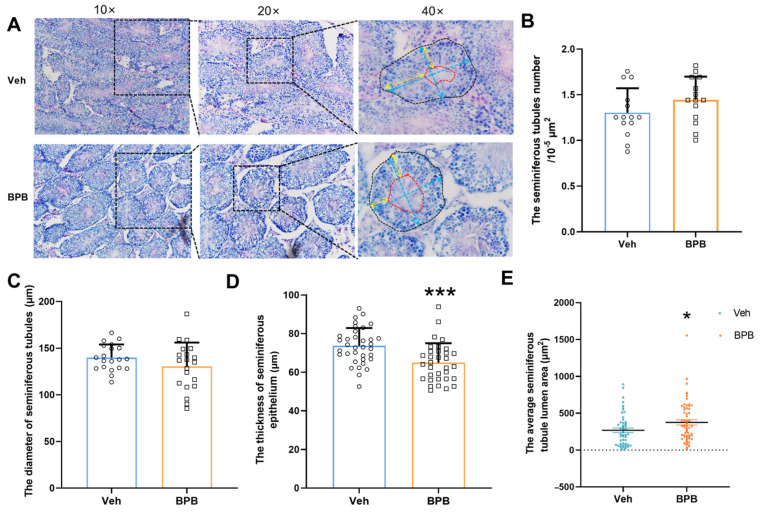
Impact of maternal BPB exposure on seminiferous tubule morphology in adult male offspring. (**A**) Representative images of H&E-stained sections. Overview images were showed at 10×, 20×, and 40× magnification. At 40× magnification, a seminiferous tubule outlined with black dashed lines. Blue lines indicate the diameter (average of two perpendicular diameters); yellow lines indicate epithelium thickness; red outline indicate the lumen area. (**B**) Count of seminiferous tubules; (**C**) mean diameter of seminiferous tubules; (**D**) mean thickness of the seminiferous epithelium; (**E**) mean lumen area. Scale bar = 50 µm. Data are shown as mean ± SEM. Data are calculated based on 21 slices from 3 mice per group. The circles represent individual data points from Veh-exposed mice and squares represent individual data points from BPB-exposed mice. * *p* < 0.05 and *** *p* < 0.001 indicate significant differences between two groups. Representative H&E-stained testis sections from adult male offspring.

To investigate whether maternal BPB exposure induces morphological abnormalities in the testis of PND 70 male offspring, we conducted H&E examination of the testicular sections (Figure 2A). The data revealed no significant changes in the number and mean diameter of seminiferous tubules in the BPB-treated group (Figure 2B,C). However, a significantly decreased thickness of seminiferous epithelium and increase in the average lumen area were observed following BPB exposure (Figure 2D,E), indicating a potential impact on spermatogenesis.

### 3.3. Maternal BPB Exposure Reduces Sperm Count and Testosterone Synthesis in Adult Male Offspring

**Figure 3:**
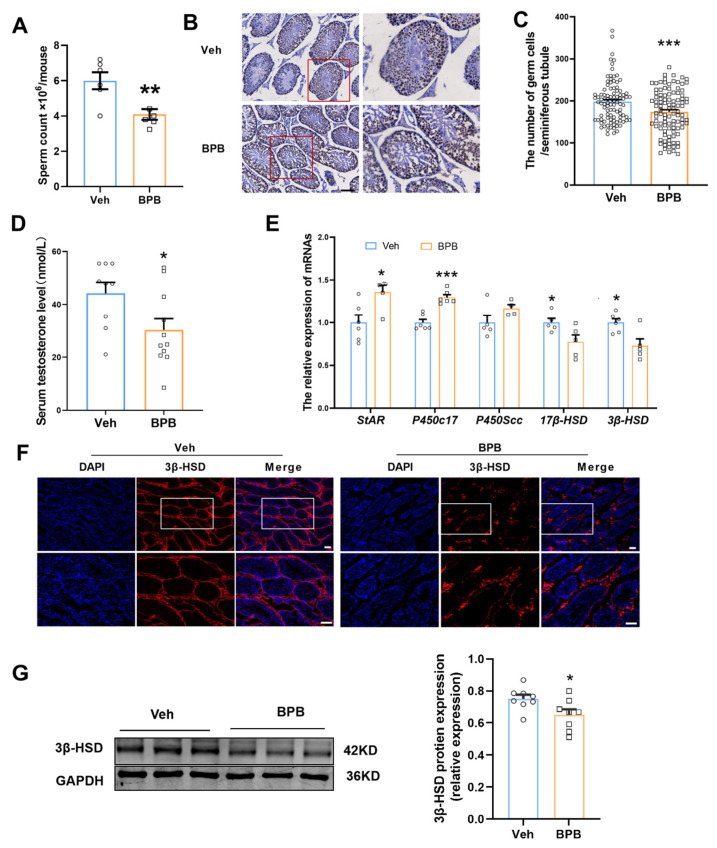
Effects of maternal BPB exposure on spermatogenesis and testosterone synthesis in adult male offspring. (**A**) Total sperm count (N = 6); (**B**) representative image of TRA98 staining, which is a marker for germ cells; (**C**) quantitative analysis of germ cell numbers (data are calculated based on 21 slices from 3 mice per group); (**D**) serum testosterone levels (N = 9–11); (**E**) mRNA expression levels of genes involved in testosterone synthesis; data are presented as mean ± SEM (N = 4–9). (**F**) Representative image of 3β-HSD staining, which is a marker for LCs (N = 3). Scale bar = 50 µm. (**G**) The protein level of 3β-HSD (N = 8). The region within the red box in the left panel was magnified at higher magnification in the right panel. The region within the white box in the top panel was magnified at higher magnification in the bottom panel. The circles represent individual data points from Veh-exposed mice and squares represent individual data points from BPB-exposed mice. * *p* < 0.05, ** *p* < 0.01 and *** *p* < 0.001 indicate significant differences between two groups.

To assess the effects of maternal BPB exposure on spermatogenesis, we evaluated sperm quantity on PND 70 and found a notable reduction in sperm count in the BPB-exposed mice (Veh: 5.99 × 10^6^/mouse, BPB: 4.08 × 10^6^/mouse) (Figure 3A). Additionally, the count of TRA98-positive germ cells, including spermatogonia, spermatocytes, spermatids, and elongated spermatids, was significantly decreased within each seminiferous tubule in the exposed group (Figure 3B,C).

Spermatogenesis and male fertility are reliant on adequate testosterone levels. To investigate whether BPB influences sperm production by disrupting the reproductive hormones, we analyzed serum testosterone levels and the expression of genes associated with hormone synthesis in the testes. We found that the mean serum testosterone level in the BPB group (30.27 ± 4.30 nmol/L) was significantly lower compared to the Veh group (44.27 ± 4.11 nmol/L) (Figure 3D). Moreover, prenatal BPB exposure altered the transcript levels of key steroidogenic enzymes, specifically reducing hydroxysteroid dehydrogenases (HSDs) *3β-HSD* and *17β-HSD*, which primarily catalyze the terminal steps of steroidogenesis, while increasing *P450c17* and *StAR*, the rate-limiting enzymes for steroidogenesis (Figure 3E). Leydig cells (LCs) are the primary steroidogenic cells responsible for testosterone production [23]. Immunofluorescence staining indicated a significant decline in LCs which were marked by 3β-HSD expression in BPB exposure compared to Veh mice (Figure 3F). Furthermore, Western blot analysis revealed that 3β-HSD protein levels in the BPB group were significantly decreased by 12% compared to the Veh group (*p* < 0.05; Figure 3G). These findings suggest that maternal BPB exposure reduces testosterone production and disrupts spermatogenesis by impairing LCs and the expression of testicular testosterone biosynthesis enzymes.

### 3.4. Maternal BPB Exposure Alters Immunological Response-Related Biological Processes in the Testis of Adult Male Offspring

**Figure 4:**
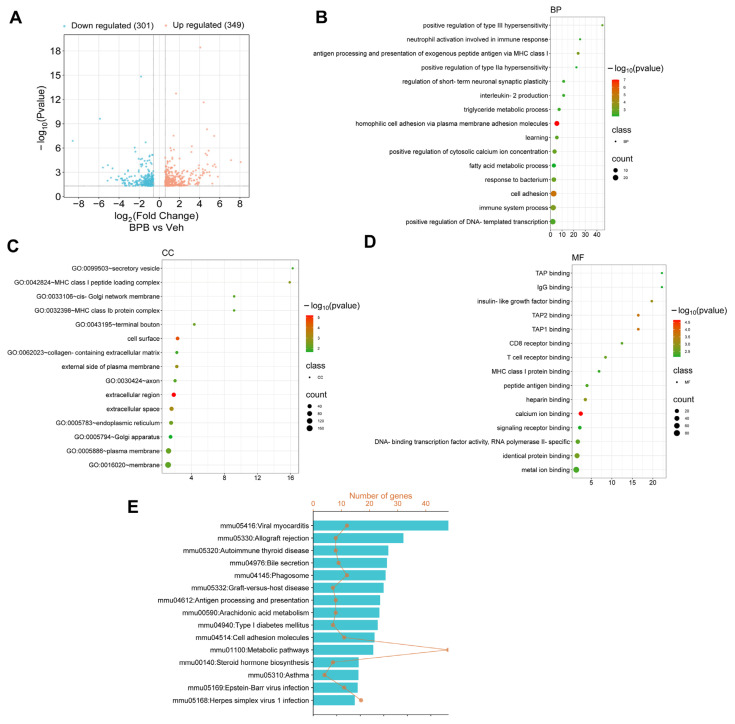
Transcriptomic effects of maternal BPB exposure on the testes of adult offspring. (**A**) Volcano plot depicting DEGs with *p*-value < 0.05 and |fold change| ≥ 1.5; (**B**) GO analysis of biological processes; (**C**) cellular components; (**D**) molecular functions; (**E**) KEGG pathway enrichment of DEGs. All GO terms and KEGG pathways with a *p*-value < 0.05 are listed in Tables S1 and S2, respectively. Abbreviations: GO, Gene Ontology; BP, biological process; CC, cellular component; MF, molecular function; DEGs, differentially expressed genes.

To determine the changes in gene expression in the testes of male offspring, we conducted deep sequencing analysis on RNA transcripts from the Veh- and BPB-exposed groups on PND 70. The data revealed 650 significantly differentially expressed genes (DEGs), of which 349 were upregulated and 301 were downregulated (Figure 4A). Gene Ontology (GO) analysis showed that these DEGs were significantly associated with several immunity-related biological processes (BPs), such as GO:0002376~immune system process, GO:0042590~antigen processing and presentation of exogenous peptide antigen via MHC class I, GO:0009617~response to bacterium, GO:0032623~interleukin-2 production, and GO:0002283~neutrophil activation involved in immune response (Figure 4B and Table S1). Additionally, adhesion molecules of immunoglobulin superfamily-related terms such as GO:0007156~homophilic cell adhesion via plasma membrane adhesion molecules and GO:0007155~cell adhesion were also significantly enriched (Figure 4B and Table S1). In the GO cellular component (CC) ontology, the DEGs showed significant enrichment in immune response-related categories, including GO:0042824~MHC class I peptide loading complex, GO:0032398~MHC class Ib protein complex, GO:0033106~cis-Golgi network membrane, and GO:0005794~Golgi apparatus (Figure 4C and Table S1). Correspondingly, in the GO molecular function (MF) terms enriched by DEGs, a majority were associated with receptor binding within the immunoglobulin superfamily, including GO:0046979~TAP2 binding, GO:0046978~TAP1 binding, GO:0042608~T cell receptor binding, GO:0042610~CD8 receptor binding, GO:0042605~peptide antigen binding, GO:0042288~MHC class I protein binding, GO:0019864~IgG binding, and GO:0046977~TAP binding (Figure 4D and Table S1). Moreover, KEGG pathway analysis demonstrated that the DEGs were significantly enriched in immune-related diseases and signaling pathways, including mmu05416: Viral myocarditis, mmu05330: Allograft rejection, mmu05320: Autoimmune thyroid disease, mmu04145: Phagosome, mmu05332: Graft-versus-host disease, mmu04612: Antigen processing and presentation, and mmu04514: Cell adhesion molecules (Figure 4E and Table S2). Collectively, these data suggest that prenatal BPB exposure may perturb testicular immune homeostasis in adult offspring.

### 3.5. Maternal BPB Exposure Induces Testicular MHC I Overexpression, Immunological Dysregulation, and ERα Downregulation in Adult Male Offspring

**Figure 5:**
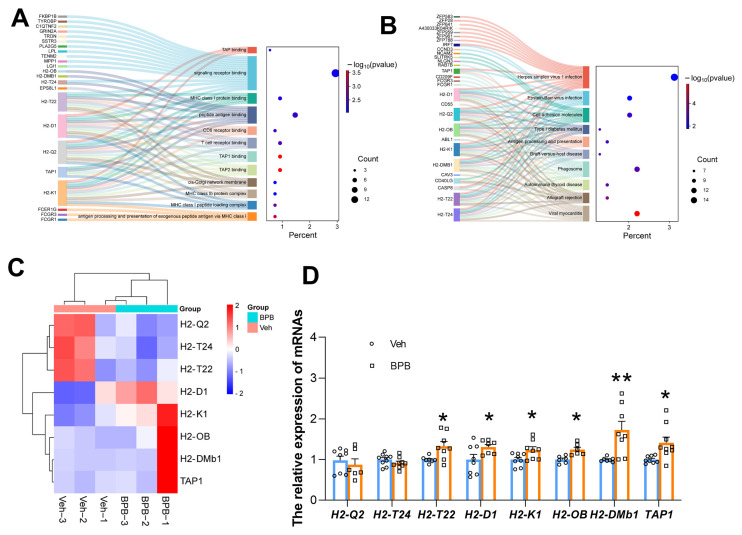
Effects of maternal BPB exposure on MHC class I molecule expression in testes of adult male offspring: (**A**) MHC class I molecules associated with immune-related GO terms; (**B**) KEGG signaling pathways involving MHC class I molecules; (**C**) heatmap of MHC class I gene expression from RNA-seq data; (**D**) qRT-PCR validation of MHC class I gene expression. Data are shown as mean ± SEM. N = 6–9. The circles represent individual data points from Veh-exposed mice and squares represent individual data points from BPB-exposed mice. * *p* < 0.05 and ** *p* < 0.01 indicate significant differences between two groups.

It is particularly noteworthy that MHC class I molecules (such as H2-T24, H2-T22, H2-OB, H2-Q2, H2-D1, and H2-K1) are predominant in nearly all immune-related GO terms, including BP, CC, and MF (Figure 5A), as well as in KEGG signaling pathways (Figure 5B). Furthermore, more than half of the genes displayed significant upregulation following exposure (Figure 5C), indicating that these molecules could contribute to the observed reproductive impairments in adult males following prenatal BPB exposure. Additionally, qRT-PCR results explicitly demonstrated that except for *H2-T24* and *H2-Q2*, other genes were significantly upregulated in the testes of adult male offspring after exposure (Figure 5D).

**Figure 6:**
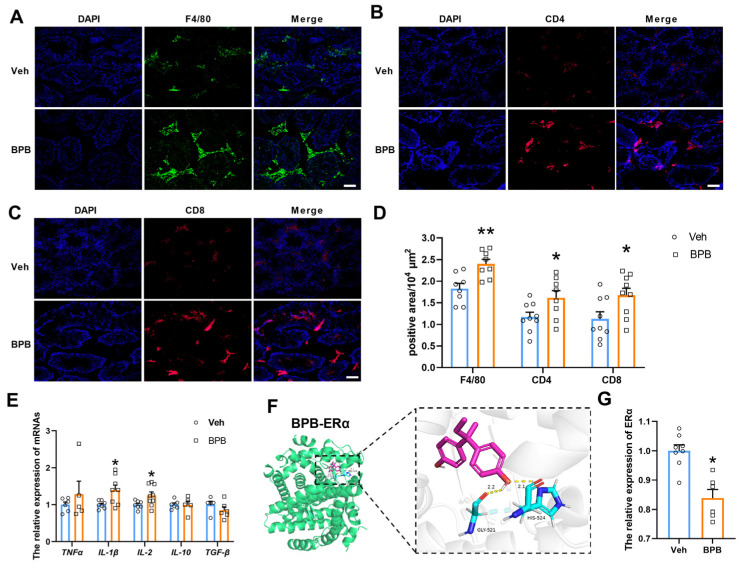
Impact of maternal BPB exposure on testicular immune responses and underlying molecular mechanisms. (**A**) Representative immunofluorescent staining images for macrophages labeled by F4/80 (green); (**B**) CD4 T cells (red); (**C**) CD8 T cells (red) in testicular sections; (**D**) the quantitative analysis of the positive staining area of F4/80, CD4, and CD8. N = 6–9 slides from 3 testes. (**E**) Relative mRNA expression of cytokines (N = 5–6); (**F**) molecular docking results and the binding mode between BPB and ERα. (**G**) Relative mRNA expression of ERα. Scale bar = 50 µm. Data are shown as mean ± SEM (N = 6–9). The circles represent individual data points from Veh-exposed mice and squares represent individual data points from BPB-exposed mice. * *p* < 0.05 and ** *p* < 0.01 indicate significant differences between two groups.

Testicular macrophages represent the largest population of immune cells within the testis and predominantly express major histocompatibility class II (MHC II) while being M2-polarized under homeostatic conditions [24]. These macrophages are essential for fetal vasculature development, LC steroidogenesis, and spermatogonial stem cell function, and they contribute to the maintenance of testicular immune privilege by skewing innate immune responses [25]. Upon stimulation, macrophages can process and present exogenous antigens on MHC class I molecules through both classical TAP (transporter associated with antigen processing)-dependent and independent pathways, subsequently activating CD8+ T cells [26]. In this study, DEGs were significantly enriched in GO categories related to TAP binding, such as GO:0046979~TAP2 binding, GO:0046978~TAP1 binding, GO:0042605~peptide antigen binding, and GO:0046977~TAP binding (Figure 4D), combined with the markedly upregulated TAP1 following exposure (Figure 5D) indicating that prenatal BPB exposure may enhance the loading of MHC class I molecules on macrophages via the TAP-dependent pathway. Concurrently, the enriched GO-MF terms, such as GO:0042608~T cell receptor binding and GO:0042610~CD8 receptor binding (Figure 4D), suggested that macrophages present exogenous antigens on MHC class I molecules potentially activating CD8+ T cells. Moreover, we observed a notable increase in the number of F4/80+ macrophages (Figure 6A) and CD4+ and CD8+ T cell populations (Figure 6B,C) in the exposed group (Figure 6D). By assessing pro-inflammatory cytokines (IL-2, TNFα, and IL-1β) and anti-inflammatory factors (IL-10 and TGF-β), the results showed that the mRNA expression levels of *IL-1β* and *IL-2* were significantly increased in the testes of BPB-exposed subjects compared to the Veh group (Figure 6E). These findings suggest that prenatal BPB exposure may contribute to testicular immunological responses and inflammation.

Studies have shown a negative correlation between MHC I expression and estrogen receptor (ER) expression [27]. Given the significant downregulation of ERα in the testes of adult offspring following prenatal BPB exposure, we hypothesize that BPB exposure may perturb ERα signaling, thereby impairing MHC I expression and ultimately driving immune dysregulation. Interestingly, molecular docking simulations predict that BPB could bind to ERα via hydrogen bonds with Gly521 and His524, suggesting a testable mechanism for ERα modulation (Figure 6F). Additionally, qRT-PCR results demonstrated that prenatal BPB exposure significantly inhibited the ERα expression in the testis of adult offspring (Figure 6F). This suggests that maternal BPB exposure may induce MHC I overexpression by suppressing ERα, leading to macrophage and T cell-mediated immune responses, which ultimately result in male reproductive damage.

## 4. Discussion

Endocrine-disrupting chemicals (EDCs) can interfere with hormonal effects in the body, disrupt normal endocrine functions, and thereby endanger the health of humans and animals [28]. Bisphenol A (BPA) is one of the most abundant EDCs [29]. It has been reported that in men, BPA exposure can reduce testosterone levels, affect sperm production and quality, and increase sperm DNA damage [30,31]. BPA exposure during the fetal period can affect endogenous long-chain fatty acid metabolism and steroid production in adult testes, which may lead to impaired sperm maturation and quality [32]. Furthermore, evidence indicates that BPA may disrupt the hypothalamic–pituitary–gonadal axis and interfere with the transcription of DNA methyltransferases (Dnmts) in male offspring testes [33], thereby impairing testosterone, estradiol, and estrogen receptor secretion [34], which damages testicular tissue and affects reproductive function. BPA can also affect female cyclicity, leading to ovarian and uterine dysfunction, including altered steroid production, follicular formation, and oocyte production [35,36]. As an alternative structurally similar to BPA, BPB has similar physical and chemical properties and has also been reported to have negative impacts on the male reproductive system. Acute and subacute exposure of adolescent male mice to BPB had adverse effects on sperm production, morphology, and function [37,38]. Adult rats receiving 50 mg/kg of BPB daily for 30 consecutive days showed induced sperm cell DNA damage and decreased sperm counts [37]. In female rats treated with BPB, the ovaries showed a significant reduction in antral follicles and corpus luteum, as well as an increase in atretic and cystic follicles [38]. One study demonstrated that by detecting testosterone levels, oxidative stress markers, and antioxidant enzyme activities in the reproductive tissues of rat testes after exposure to 5, 25, and 50 mg/kg/day of BPA and its analogs BPB, BPF, and BPS for 28 days, these compounds exhibited comparable effects [38]. Additionally, Qiu et al. discovered that during the embryonic and larval development of zebrafish, exposure to 100 μg/L of BPA or its analogs, including BPB, increased the expression of reproductive neuroendocrine-related genes and the levels of typical hormones such as LH, FSH, E2, and GH at 120 hpf. However, the intensity of gene expression promoted by BPB was lower than that induced by BPA [39]. As a BPA alternative, BPAF exposure induces H3K27me3 hypermethylation in testicular tissue and disrupts the cytoskeletal structure and tight junction permeability of Sertoli cells by activating the ERK signaling pathway to regulate actin regulatory proteins (e.g., Arp3 and Palladin), thereby interfering with the specialized microenvironment created by Sertoli cells for spermatogenesis [40]. Given that BPB shares estrogenic effects similar to BPA and BPAF, we hypothesize that BPB may mediate male reproductive dysfunction through analogous mechanisms.

In this study, the data reveal that prenatal BPB exposure disrupts the development of seminal vesicles and preputial glands, enlarges the average testicular lumen area, and decreases sperm count and testosterone synthesis. Notably, functional enrichment analysis of significant DEGs highlighted the abnormal expression of Leydig cell- and immune-related genes, suggesting that prenatal BPB exposure may primarily lead to testicular dysfunction through immune activation. Additionally, we confirmed that BPB induces MHC I overexpression by inhibiting *ERα* in the offspring’s testes, which increases inflammatory factor expression and elicits CD4+ and CD8+ T cell responses. This finding aligns with our previous results on the reproductive developmental toxicity of BPAF [41]. These findings offer new insights into the mechanisms underlying male reproductive disorders caused by prenatal BPB exposure and suggest potential therapeutic targets. Future studies should explore additional mechanisms (e.g., oxidative stress, apoptosis, and epigenetic dysregulation) underlying prenatal BPB-induced male reproductive abnormalities.

It is well-established that the regulation of male reproductive function and spermatogenesis is strongly associated with the modulation of estrogenic, androgenic, and steroidogenic endocrine activities. BPB has been demonstrated to mimic estrogen activity, binding competitively to the ER in a variety of species, including humans, rats, and mice. This interaction allows BPB to modulate gene expression by engaging with either or both of the estrogen receptor subtypes, ERα and ERβ [4]. However, limited studies have shown that BPB exerts positive effects on ERα-mediated transcriptional activation, which is associated with the modulation of ERα expression, particularly in testicular tissues. A dose–response investigation revealed that one of the bisphenols, BPAF, acts as an antagonist of transcriptional activities via ERE activation, with effects ranging from minimal at 5 µM to maximal at 25 µM, demonstrating anti-estrogenic effects in ERα-positive MCF-7 cells. Here, molecular docking simulations predict that BPB could bind to ERα, while prenatal exposure significantly decreased its expression in the testes of adult male offspring. These predictive findings require further experimental validation through competitive binding assays or ERα mutant models. The observed reproductive dysfunction may be attributed to the downregulation of ERα itself. Our results are consistent with a study in MCF-7 cells, which showed that although no significant reduction in ERα levels was observed following BPB exposure, there was a tendency for negative modulation of ERα to some extent [42]. Considering the widespread distribution of ERα in the testis and its role in spermatogenesis (including proliferation, apoptosis, survival, and maturation) [43], our results indicate that prenatal BPB exposure leads to male reproductive dysfunction by inhibiting ERα expression.

The adverse effects of prenatal BPB exposure on adult testicular function are likely mediated through indirect mechanisms. Given that BPB is a structural analog of BPA, it may interfere with fetal steroidogenesis, thereby altering the developmental trajectory of testicular niches. BPB has been reported to exhibit steroidogenesis-related endocrine activity, including effects on enzyme activity, steroid hormone precursors, steroidogenesis-related gene expression. Studies using H295R cells have demonstrated that exposure to BPB at low micromolar concentrations results in decreased levels of androstenedione, testosterone, and cortisol, as well as an increase in estrogen level [44]. In vitro studies revealed that Cyp19a1 gene expression was reduced following BPB exposure, though this effect was observed only at relatively high concentrations (IC50 of 44.27 μM) [42]. Desdoits-Lethimonier et al. found that exposed human testicular tissue samples with BPB for 24/48 h resulted in variable decreases in testosterone production [45]. Here, our results demonstrated that prenatal BPB exposure obviously decreased testosterone levels, downregulated the expression of testicular testosterone biosynthesis enzymes, and reduced sperm count in adult male offspring. These findings further confirm that BPB exposure, even indirectly, affects endocrine activity related to steroidogenesis, which consequently alters steroidogenesis in the testis.

Testicular macrophages are the main immune cell type in the mammalian testis and are crucial for maintaining testicular immune privilege by modulating innate immune responses. These cells are essential for supporting testicular processes like fetal vasculature development, LC steroidogenesis, and spermatogonial stem cell activity. Macrophages present foreign antigens using MHC classes I and II and are considered antigen-presenting cells. Under steady-state conditions, interstitial macrophages near LCs and blood vessels express high levels of antigen presentation genes such as MHC class II. Conversely, peritubular macrophages near the seminiferous tubules have high levels of immunosuppressive genes, IL10 and Marco [46]. Maternal BPB exposure significantly increased macrophages in the adult offspring testis, with notable upregulation of MHC class I genes, indicating a strong reaction disrupting the normally suppressed macrophage immune response. Macrophages present exogenous antigens using MHC class I molecules to activate CD8+ T cells [47]. CD8+ T cells were increased in the exposed testes, suggesting that maternal BPB exposure disrupts the testicular immune microenvironment and triggers both innate and adaptive immune responses. Immune cells are crucial for the cellular niche required for steroid hormone production. Research over the past decades shows that testicular macrophages and LCs are interrelated and rely on each other for testis development [46]. In inflammation, activated testicular macrophages release factors that inhibit LC steroidogenesis and impair testicular function [48]. Taken together, prenatal BPB exposure may decrease testosterone production and impair spermatogenesis by activating the testicular immune response.

Research indicates that cytokine expression and macrophage activities can be modulated by estrogens and their receptors. MHC I expression has been reported to be inversely related to ER expression. For instance, HLA-ABC protein levels decreased following β-estradiol treatment or hESR-GFP transfection and increased following fulvestrant or IFN-γ treatment in cell lines [27]. Additionally, estrogens influence macrophage polarization through ERα, promoting a shift toward a more M2-like phenotype, while ERα ablation prevents this polarization [49]. Another study showed that activating a macrophage-specific E2/ER signaling axis enhances M2 polarization and suppresses CD8+ T-cell function in the melanoma tumor microenvironment [50]. G protein-coupled estrogen receptor 1 (GPER1), a novel estrogen receptor, modulates macrophage activities and the pro-inflammatory pathway [51]. Considering the increased levels of pro-inflammatory factors and MHC I class genes, along with the decreased expression of ERα in the exposed group, our results suggest that prenatal BPB exposure elicits the MHC I-mediated immunological response in the testis of adult male offspring, primarily due to the inhibition of ERα signaling. However, some limitations need to be addressed in the future as follows: (1) follow-up investigations should incorporate graded dose groups (prioritizing human-relevant low-exposure ranges) to systematically evaluate dose-dependent phenotypic effects. (2) The potential roles of Sertoli cell dysfunction and alternative pathways require further exploration. (3) While molecular docking predicts ERα-binding interactions, experimental validation through competitive binding assays remains essential.

## 5. Conclusions

Maternal BPB exposure adversely affects male reproductive health through indirect mechanisms, causing significant disruptions in reproductive organ development, testicular morphology, testosterone level, and sperm production. Specifically, BPB may bind to ERα, and prenatal BPB exposure significantly downregulates ERα expression, leading to the upregulation of MHC I-related genes, increased inflammatory factors, and promoting the activation of macrophage, CD4+, and CD8+ T cells, ultimately disrupting testosterone biosynthesis and impairing spermatogenesis. These findings enhance our understanding of the mechanisms underlying male reproductive disorders associated with prenatal BPB exposure and highlight potential targets for therapeutic intervention.

## Figures and Tables

**Table 1:** The primers used in the quantitative real-time PCR.

Gene Name	Forward Primer	Reverse Primer
*H2-T24* (histocompatibility 2, T region locus 24)	GTCGCACTCTCTGCATTACTG	CTGCTGTCGTAGTGGATGAAG
*H2-T22* (histocompatibility 2, T region locus 22)	GCCTTGGATTTGGATTGTTGC	AAGACTCGCCAACTGAAGTTC
*H2-DMB1* (histocompatibility 2, class II, locus Mb1)	ACCCCACAGGACTTCACATAC	GGATACAGCACCCCAAATTCA
*H2-K1* (histocompatibility 2, K1, K region)	CGTTCCAGGGGATGTACGG	GCTCCCACTTGTGTTTGGTGA
*H2-OB* (histocompatibility 2, O region beta locus)	AGGCGGACTGTTACTTCACC	ATCCAGGCGTTTGTTCCACTG
*TAP1* (transporter 1, ATP binding cassette subfamily B member)	GGACTTGCCTTGTTCCGAGAG	GCTGCCACATAACTGATAGCGA
*H2-Q2* (histocompatibility 2, Q region locus 2)	ACGCGGAGAAACCGAGGTA	CCGTCCGATCCCACATCAC
*H2-D1* (histocompatibility 2, D region locus 1)	TGGTGCTGCAGAGCATTACA	TGTGCCTTTGGGGAATCTGT
*ERα* (estrogen receptor alpha)	TTGAACCAGCAGGGTGGC	AGGCTTTGGTGTGAAGGGTC
*TNFα* (tumor necrosis factor alpha-like)	CATCTTCTCAAAATTCGAGTGACAA	TGGGAGTAGACAAGGTACAACCC
*IL-10* (interleukin 10)	CTTACTGACTGGCATGAGGATCA	GCAGCTCTAGGAGCATGTGG
*IL-1β* (interleukin 1 beta)	CAACCAAAAGTGATATTCTCCATG	GATCCACACTCTCCAGCTGCA
*TGF-β* (transforming growth factor, beta receptor I)	CCACCTGCAAGACCATCGAC	CTGGCGAGCCTTAGTTTGGAC
*IL-2* (interleukin 2)	ACACCTTTAATTGGTCAACACGA	CCTGCTACGTTCTCTACCTCT
*Gapdh*	AGGTCGGTGTGAACGGATTTG	TGTAGACCATGTAGTTGAGGTCA

## Data Availability

The data supporting the conclusions of this article are available from the corresponding author upon reasonable request.

## Supplementary Materials

The following supporting information can be downloaded at: https://www.mdpi.com/article/10.3390/toxics13060423/s1, Figure S1: Chemical structures of BPA and BPB. Table S1. GO enrichment of DEGs. Table S2. KEGG pathway enrichment of DEGs.
