# Supplementary material for: Prenatal Bisphenol B Exposure Induces Adult Male Offspring Reproductive Dysfunction via ERα Inhibition-Triggered MHC I-Mediated Testicular Immunological Responses
Source: Toxics. 2025 May 22;13(6):423. doi: 10.3390/toxics13060423 (PMC12197064; doi:10.3390/toxics13060423)
Supplement: Supplementary file 1 [file toxics-13-00423-s001.zip › Supporting Information.pdf]

Supporting Information for

Prenatal Bisphenol B Exposure Induces Adult Male Offspring Reproductive Dysfunction via ERα Inhibition-Triggered MHC I-Mediated Testicular Immunological Responses

**Nannan Chen [1,†], Xiaotian Li [1,†], Shenrui Zhou [1], Xin Peng [1], Senlin Xue [1,2], Yuetong Liu [3], Tingwang Jiang [2]
and Wei Yan [1,*]**

[1] School of Life Science, Xuzhou Medical University, Xuzhou 221004, China; 303112111087@stu.xzhmu.edu.cn (N.C.); 202123010127@stu.xzhmu.edu.cn (X.L.); 202123010121@stu.xzhmu.edu.cn (S.Z.); 202123010105@stu.xzhmu.edu.cn (X.P.); xuesenlin2020@163.com (S.X.)

[2] Department of Key Laboratory, Affiliated Changshu Hospital of Nantong University,
Changshu 215500, China; 5301002@ntu.edu.cn

[3] School of Nursing, Xuzhou Medical University, Xuzhou 221004, China; 202304011321@stu.xzhmu.edu.cn

* Correspondence: yanwei@xzhmu.edu.cn; Tel.: +86-0516-83262079

† These authors contributed equally to this work.

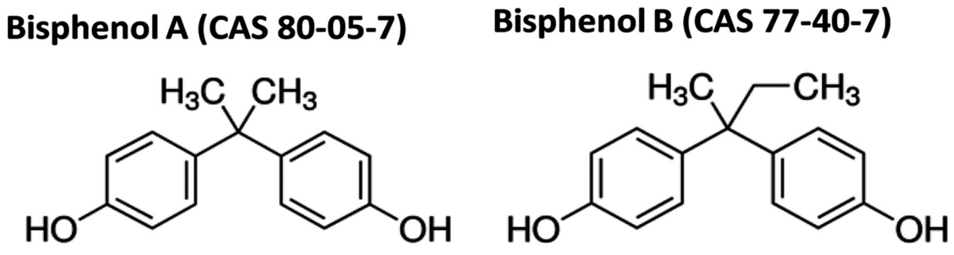

Figure S1. Chemical structures of BPA and BPB.
